# Whole exome sequencing reveals pathogenic variants in *MYO3A*, *MYO15A* and *COL9A3* and differential frequencies in ancestral alleles in hearing impairment genes among individuals from Cameroon

**DOI:** 10.1093/hmg/ddaa225

**Published:** 2020-10-20

**Authors:** Ambroise Wonkam, Noluthando Manyisa, Christian D Bope, Collet Dandara, Emile R Chimusa

**Affiliations:** Division of Human Genetics, Department of Pathology, University of Cape Town, Cape Town 7925, South Africa; Department of Medicine, Faculty of Health Sciences, University of Cape Town, Cape Town 7925, South Africa; Division of Human Genetics, Department of Pathology, University of Cape Town, Cape Town 7925, South Africa; Department of Mathematics and Department of Computer Science, Faculty of Sciences, University of Kinshasa, Kinshasa, Democratic Republic of Congo; Division of Human Genetics, Department of Pathology, University of Cape Town, Cape Town 7925, South Africa; Division of Human Genetics, Department of Pathology, University of Cape Town, Cape Town 7925, South Africa

## Abstract

There is scarcity of known gene variants of hearing impairment (HI) in African populations. This knowledge deficit is ultimately affecting the development of genetic diagnoses. We used whole exome sequencing to investigate gene variants, pathways of interactive genes and the fractions of ancestral overderived alleles for 159 HI genes among 18 Cameroonian patients with non-syndromic HI (NSHI) and 129 ethnically matched controls. Pathogenic and likely pathogenic (PLP) variants were found in *MYO3A, MYO15A* and *COL9A3*, with a resolution rate of 50% (9/18 patients). The study identified significant genetic differentiation in novel population-specific gene variants at *FOXD4L2, DHRS2L6, RPL3L* and *VTN* between HI patients and controls. These gene variants are found in functional/co-expressed interactive networks with other known HI-associated genes and in the same pathways with *VTN* being a hub protein, that is, focal adhesion pathway and regulation of the actin cytoskeleton (*P*-values <0.05). The results suggest that these novel population-specific gene variants are possible modifiers of the HI phenotypes. We found a high proportion of ancestral allele versus derived at low HI patients-specific minor allele frequency in the range of 0.0–0.1. The results showed a relatively low pickup rate of PLP variants in known genes in this group of Cameroonian patients with NSHI. In addition, findings may signal an evolutionary enrichment of some variants of HI genes in patients, as the result of polygenic adaptation, and suggest the possibility of multigenic influence on the phenotype of congenital HI, which deserves further investigations.

## Introduction

Hearing impairment (HI) is a common sensory impairment, affecting nearly 500 million people worldwide (1). The burden of HI has worsened globally and is the leading cause of disability (2), with a projected increasing trend (3)*.* More than 80% of people affected by HI live in low- and middle-income countries. The highest incidence rate is found in the sub-Saharan Africa (SSA): up to 6 per 1000 compared with about 1 per 1000 in Europeans or North Americans (4)*.*

**Table 1:** Demographics of patients with HI and controls

Demographics	Categories	*n* (%) Patients	*N* (%) Controls
Sex	Male	10 (52·6)	53 (41·1)
	Female	8 (47·4)	73 (56·6)
	Blank		3 (2·3)
Age of onset	Congenital	17 (89·5)	
	Prelingual	2 (10·5)	
	Blank		129 (100·0)
Degree of HI left	Severe	1 (5·3)	
	Profound	17 (89·4)	
	Blank^a^	1 (5·3)	129 (100·0)
Degree of HI right	Severe	2 (10·5)	
	Profound	16 (84·2)	
	Blank^a^	1 (5·3)	129 (100·0)
Transmission	Autosomal recessive	18 (100)	
	Not applicable		129 (100·0)

^a^No data available.

**Table 2:** Candidate variants associated with HI in Cameroonian patients

No. of patients (age in years)	Family pedigree	Genotype	Gene	Region	SNP	cDNA change	Protein change	Functional effect^a^	ExAC_AFR MAF	ExAC_ASI MAF	Cameroonian controls MAF (*N* = 129)
3 (10, 10, 16)	SP, SP, MP	Homozygous	*MYO3A*	10p12.1	rs140301218	c.C424T	p.H142Y	Loss of function of protein kinase domain	0.0026	0.0007	0
4 (12, 8, 10, 10)	MP, MP, SP, MP	Homozygous	*MYO15A*	17p11.2	rs138861831	c.4888 T	p.R1630C	Loss of function of myosin head-like domain	0.0083	0.0003	0
2 (16, 11)	SP, MP	Homozygous	*COL9A3*	20q13.33	rs145035835	c.G406A	p.G136S	Loss of function of the triple-helical region 3	0	0.001	0

^a^Exonic non-synonymous variants that were considered deleterious/damaging according to 14 different functional scores of annotation databases, including SIFT, Polyphen2_HDIV, Polyphen2_HVAR, likelihood ratio test, MutationTaster, MutationAssessor, FATHMM, fathmm-MKL, RadialSVM, LR, CADD, PROVEAN, MetaSVM and MetaLR

Abbreviations: SP, simplex; MP, multiplex family; SNP, single nucleotide polymorphism; ExAC, exome aggregation consortium; AFR, African; AS, Asian; MAF, minor allele frequency

HI may be due to genetic, environmental and/or unknown factors. About 50% of congenital HI cases in high-income countries are due to genetic causes (5). The overshadowing environmental factors as causes of HI in SSA has meant that searching for genetic causes has not been prioritized (6–9). For example, in Cameroon, in a total of 582 patients with HI, with limited molecular investigations, the environmental factors were estimated at 52.6%, the genetic causes at 14.8% and unknown causes, some of which could be genetic, were estimated at 32.6% (8). Non-syndromic HI (NSHI) accounts for approximately 70% of genetic HI (5) and is described as the condition where HI occurs without any additional clinical manifestations. Autosomal recessive inheritance accounts for the majority (77–80%) of NSHI (5,10,11).

Variants in more than 150 genes have been reported as possible causes of HI (12). Mutations in the connexin genes *GJB2* and *GJB6* have been shown to account for most of genetic-associated HI among European, Asian and North American populations (13–15). However, a few studies investigated the genetic cause of HI among Africans (7,9,16–22), with most of them showing that *GJB2* and *GJB6* may not always be the important contributors, with the exception of Ghana, where the *GJB2*-R143W founder mutation accounts for 25·9% of HI cases among families segregating autosomal recessive NSHI (ARNSHI) (23).

Advanced genomic technologies, particularly targeted enrichment genome analysis by next-generation sequencing (NGS), have now made it possible for efficient determination of other genetic contributors without necessarily understanding the pathways involved. However, NGS has demonstrated a consistently low pickup rate for isolated cases of HI in individuals of African ancestry (24,25). This pickup rate was slightly improved in a modest group of multiplex families segregating HI from Cameroon (26). Hence, there is a great scarcity in the representation of known pathogenic gene variants of HI in populations of African ancestry. Indeed, a recent study of pathogenic and likely pathogenic (PLP) ARNSHI variants (selected from the ClinVar and Deafness Variation databases with their frequencies from gnomAD database) estimated the prevalence of HI due to PLP as 5·2 per 100 000 individuals for Africans, compared with a highest prevalence of 96·9 per 100 000 individuals for Ashkenazi Jews (27). The knowledge deficit of genetic causes of HI among African populations is likely hindering our current understanding of the mechanism of HI, the refinement of gene–disease pairs and clinical validity curation and is ultimately affecting the development of genetic diagnoses and counseling, therapeutics and prognosis. The efficiency of using whole exome sequencing (WES) in revealing PLP and in exploring conservative evolution in HI genes in sub-Saharan Africans has been rarely investigated.

This study used WES to first identify *in silico* PLP variants and then examine the fractions of ancestral to derived alleles for 159 known HI-associated genes (Supplementary Material, Table S1) implicated in a group of Cameroonian patients with NSHI and controls with the same ethnolinguistic background. Our study identifies PLP variants in known genes among patients and reveals that rare variants within known HI-associated genes may have evolved conservatively to play critical roles in HI phenotypic variation among African HI patients, suggesting the possibility of multigenic and polygenic adaptation influencing congenital HI.

## Results

### Patients’ information and variant discovery

The demographics of patients and controls are summarized in Table 1. There was an almost equal gender distribution of patients within this study (52·6% of study participants were male). The mean age was 12 years (±4·4) in the patients’ population and 26 years (±13·1) for the controls group. All the patients presented with non-syndromic congenital/prelingual onset of HI, with the majority (nearly 90%) having a profound HI (91 dB and above).

Our results from WES variants calling yielded 7 158 983 variants that passed quality control (Supplementary Material, Fig. S1) of which 80 226 were exonic single nucleotide polymorphisms (SNPs), distributed as 3·6% stop loss, 2·4% stop gain, 4·4% synonymous and 94% non-synonymous or splice site variants.

### *In silico* putative deleterious variants in known genes among HI patients

Upon filtering variants, results indicated PLP variants in 9/18 patients (Table 2).

In addition, putative PLP variants in *USH2A*, *HSD17B4* and *MYO1A* were identified in various patients (Supplementary Material, Table S2), but careful consideration of phenotype to genotype associations, in addition to the recent expert curation of the clinical validity of 164 HI gene–disease pairs by Clinical Genome (ClinGen) (28), allowed us to exclude them as potential candidates. Indeed, the variant found in *USH2A* (MIM:276901) is associated with Usher syndrome type IIA and was defined as benign; *HSD17B4* (MIM:601860) is associated with Perrault syndrome (MIM:233400) that has a clinical presentation not found in the patients; while *MYO1A* has been excluded as a HI-associated gene (28).

Considering all HI patients and controls together, we identified novel population-specific candidate variants in four genes: *FOXD4L6, DHRS4L2, RPL3L* and *VTN* (Supplementary Material, Table S3), which were not previously associated with HI or any other human conditions.

### Population structure

**Figure 1:**
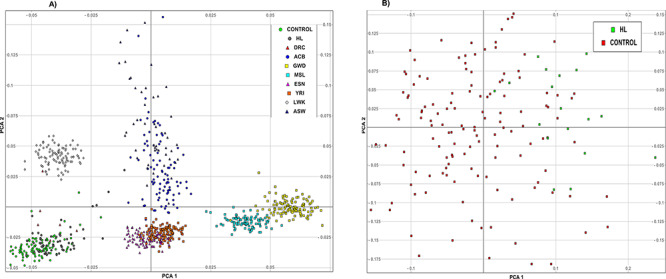
PCA plot for nine African populations. (**A**) The confirmation of the origin of the control and patient groups from the same ethnolinguistic background, which cluster in the lower left corner of the plot and are represented by the green and gray circles for the controls and patients groups, respectively. The inherent diversity of the individuals within the patients and controls groups is represented in (**B**), where the red squares indicate the controls group and the green squares represent the HI patients group. DRC: Demographic Republic of Congo (red triangle); ABC: African Caribbean in Barbados (blue circle); GWD: Gambian in Western Division –Mandinka (yellow scare); MLS: Mende in Sierra Leone (light blue square); ESN: Esan in Nigeria (purple triangle); YRI: Yoruba in Ibadan, Nigeria (orange square); LWK: Luhya in Webuye, Kenya (grey diamond); ASW: African Ancestry in Southwest United States (blue triangle).

Principal component analysis (PCA) was performed with data from Cameroonian controls and HI patients along with those of eight other African populations (Fig. 1). Cameroonian control and patient groups cluster together in the lower left corner of the plot in Figure 1A, confirming some level of population homogeneity. Regarding the second PCA, Figure 1B represents data from Cameroonian controls and patients and indicates the inherent diversity within this population.

### Genetic differentiation between Cameroon hearing-impaired patients and controls

Following WES, aggregated SNP frequencies within 159 known HI genes were analyzed for unusual differences between the patients and controls using Fisher’s combined probability (see Materials and Methods). The significance level for difference between cases and controls had a *P*-value of <0·05/159, which is 3·1 × 10^–4^, and the permutation adjusted *P*-value was also obtained after 100 000 permutations. We investigated frequencies difference in population-specific gene variants, using an approach that is similar to gene burden analysis (29), a well-calibrated approach that accounts for small-to-moderate sample size and rare variants. The results are summarized in Table 3, where up to 26 genes were identified for the differences between patients and controls, indicating potential genetic differentiation (*P*-value < 0·05).

Importantly, two identified *in silico* candidate variants in Supplementary Material, Table S2, including *RPL3L* (*P*-value *=* 3·1e-02) and *VTN* (*P*-value *=* 6·1e-04), additionally indicated genetic differentiation between HI patients and controls (Table 3)*.* These gene variants have not been previously associated with HI. The samples’ quality at these gene regions was checked back in the Binary Alignment Map (BAM) files (Supplementary Material, Fig. S2).

**Table 3:** Genetic difference between gene variants in the hearing-impaired patients and controls

Gene	Gene-specific frequency HI	Gene-specific frequency CONTROL	*P*-values	Adjusted *P*-value
***PRDX4***	0·2109	0·2618	2·65e-31	1·17e-08
***PRKACA***	0·2716	0·3211	9·71e-30	4·34e-08
***CBX1***	0.2242	0·2726	1·83e-28	9·07e-08
***COPS5***	0·2908	0·2488	4·35e-22	1·07e-07
***PRKCB***	0·2540	0·2916	4·52e-18	2·47e-07
***GLUD1***	0·2251	0·2538	2·36e-11	3·77e-06
***CRADD***	0·2571	0·2853	4·66e-11	2·67e-06
***ITGB6***	0·2426	0·2687	1·11e-09	3·47e-04
***CKS2***	0·2500	0·2753	3·40e-09	1·03e-04
***FGF2***	0·2376	0·2623	8·16e-09	1·11e-04
***IGFBP5***	0·2561	0·2799	2·68e-08	8·17e-04
***RHOXF1***	0·2655	0·2422	4·51e-08	7·27e-04
***ITGA8***	0·2559	0·2770	6·81e-07	4·47e-04
***AMPH***	0·2404	0·2606	1·91e-06	6·17e-04
***PVR***	0·2336	0·2515	2·43e-05	4·27e-03
***SERPINE1***	0·2513	0·2360	3·01e-04	2·17e-03
***RPL13A***	0·2585	0·2730	5·66e-04	1·07e-03
***VTN***	0·2601	0·2457	6·14e-04	1·11e-03
***ADH5***	0·2346	0·2209	1·16e-03	2·12e-02
***ADTRP***	0·2690	0·2816	2·77e-03	2·37e-02
***TGFB1***	0·2553	0·2428	2·84e-03	4·47e-02
***PRKCA***	0·2598	0·2710	7·42e-03	3·17e-02
***RPL3L***	0·2577	0·2487	3·13e-02	1·01e-01
***G3BP2***	0·2306	0·2396	3·23e-02	1·07e-01
***ETV7***	0·2570	0·2657	3·84e-02	1·09e-01
***PLAUR***	0·2668	0·2593	7·45e-02	1·17e-01

Genes that have been previously associated with HI. All genes above the red line have a significant genetic difference between patients and controls. Table indicates aggregated frequencies for SNPs in each gene. *P*-value was assessed using Fisher’s combined probability

**Figure 2:**
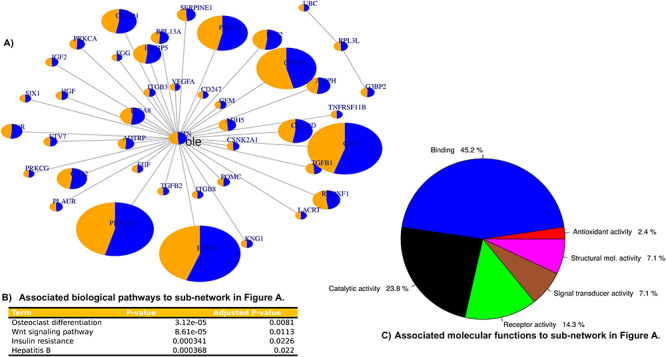
Reconstituted HI PPI network including enrichment analysis of the associated pathways and molecular functions. Figure 2 indicates PPI associated with *VTN*, *RPL3L*, *FOXD4L6* and *DHRS4L2* as well as the associated pathways and molecular functions*.* The protein products of *VTN* and *RPL3L* are associated with 42 pairings/interactions. *FOXD4L6* and *DHRS4L2* have no interactions with other HI-associated proteins, as indicated in (**A**). The interactions were queried in the Enrichr database that indicated the major pathways associated, which includes osteoclast differentiation, as indicated in (**B**). (**C**) The associated molecular function, which includes binding and catalytic activities.

**Figure 3:**
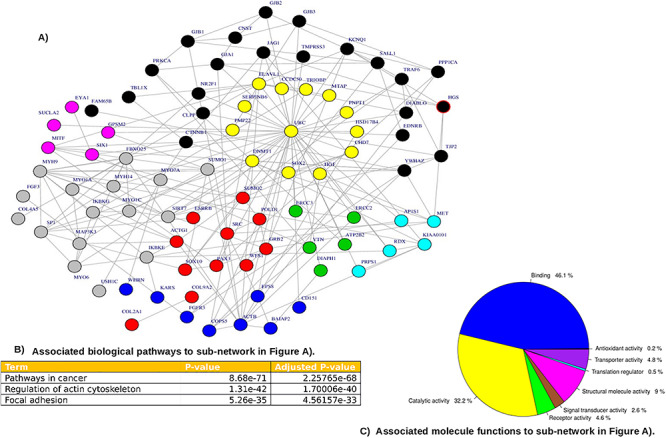
Community network analysis of the combined HI associated proteins (*n* = 159) and the population gene variant products (i.e. *VTN*, *RPL3L*, *FOXD4L6* and *DHRS4L2*)**.** The network in (**A**) indicates eight subnetworks, centered around *UBC*, with 10 hub proteins including *UBC*. The network is associated with regulation of the actin cytoskeleton and focal adhesion pathways (**B**) and with binding and catalytic activity molecular functions (**C**).

**Figure 4:**
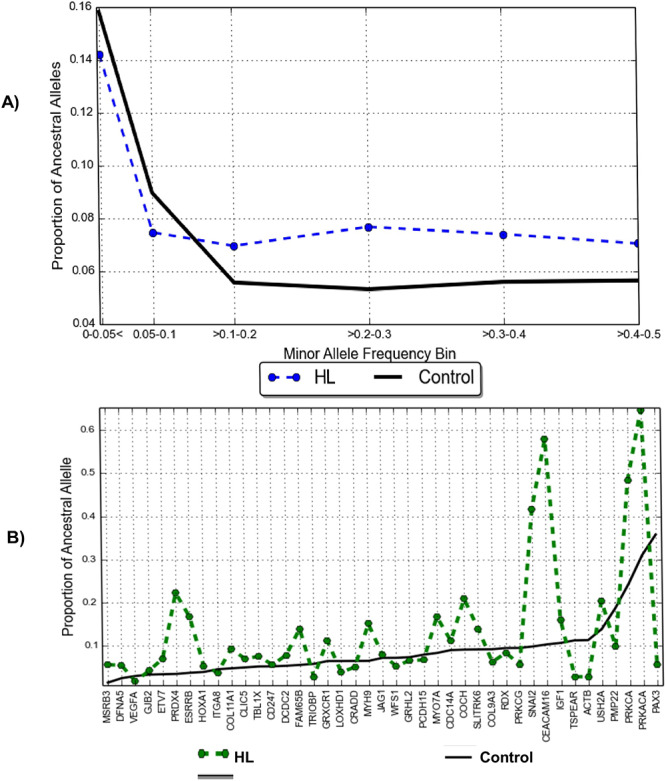
Proportion of ancestral alleles in the Cameroonian patients with HI and controls group. (**A**) Analysis indicates a higher proportion of ancestral alleles for rare variants (MAF of 0·00–0·1) in the controls groups as compared with the patients group. (**B**) Distribution of ancestral alleles proportion based on known HI genes. HL: Hearing Loss.

**Figure 5:**
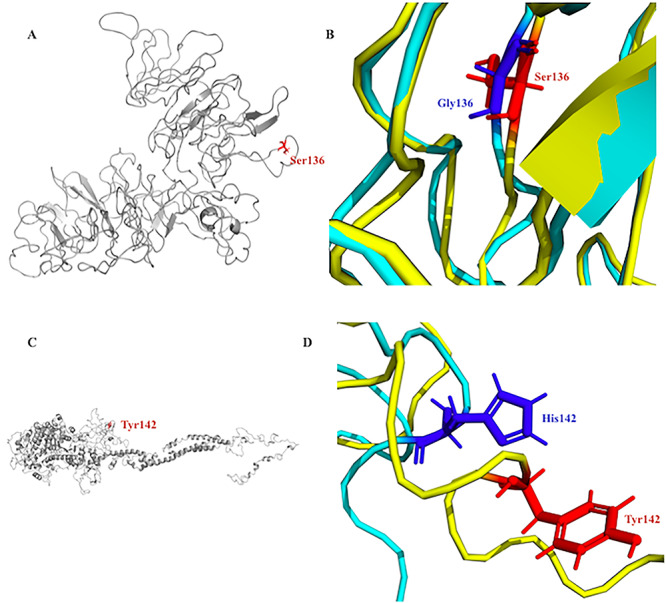
Molecular modeling scheme of the COL9A p.Gly136Ser and MYO3A p.His142Tyr mutants. (**A**) COL9A protein with mutated residue (SER136) colored red. The non-conservative substitution of uncharged non-polar GLY136 amino acid with uncharged polar amino acid SER136 renders the protein structure inflexible and may impact the binding interactions, stability and conformation of the protein. (**B**) Enlargement of the COL9A mutation site comparing the configuration of the wild type (colored cyan) and mutant (colored yellow) protein and illustrating the flexibility of the structures. (**C**) MYO3A protein product showing the mutated residue (TYR142) colored red. The substitution of the positively charged and hydrophilic amino acid HIS142 with the uncharged polar and hydrophobic amino acid TRP142 could impact the binding interactions and the stability of structure and conformation of the protein. (**D**) Enlargement of the MYO3A mutation site comparing the configuration of the wild type (colored cyan) and mutant (colored yellow) protein and illustrating the flexibility of the structures.

### Protein–protein interactions and community network enrichment analysis

The four identified genes (*VTN, RPL3L, FOXD4L6* and *DHRS2L6*) with population-specific candidate variants were queried for their functional or co-expressed interactive subnetwork, and therefore, the associated pathways and molecular functions of the resulting subnetwork. The protein products of *VTN* and *RPL3L* are indicated to functionally interact in 42 other protein–protein pairings (Fig. 2). The subnetwork in Figure 2A was shown to be significantly associated with four pathways including *osteoclast differentiation* (*P*-value = 3.2e-05), the networks interactions revealed major associated pathways, which notably included *osteoclast differentiation*  Figure 2B or catalytic activity Figure 2C.

Moreover, the interactions of the protein products of *VTN*, *RPL3L*, *FOXD4L6* and *DHRS2L6* with the other 159 HI-associated genes (Supplementary Material, Table S1) were investigated using a community network analysis. The analysis was performed by combining the candidate genes with the 159 known HI-associated genes present on the ClinVar database (30) and the Deaf Variation database (12). *VTN* is a hub protein for one of the HI-associated pathways along with nine other genes. The community network analysis provided eight protein subnetworks, which had 10 hub proteins, as indicated in Figure 3A. The associated pathways and molecular functions are indicated in Figures 3B and C, respectively. Five of the 10 hub proteins, *ACTB*, *MITF*, *SSCTG1*, *MYH9* and *ATP2B2*, are known HI-associated proteins, whereas the other hub proteins, *UBC*, *SRC*, *YWHAZ*, *KIAA010* and *VTN*, have not yet been implicated in HI. The entire community network is associated with the *focal adhesion pathway* (*P-*value = 5·3e-35) *and regulation of the actin cytoskeleton* (*P-value* = 1·3e-42), as indicated in Figure 3B. The abundant molecular processes associated with the network are *binding and catalytic activity*, as indicated in Figure 3C.

### Molecular dynamic simulations for mutated variants

Molecular dynamic (MD) simulations (Fig. 4) showed that for *COL9A*, substitution of the uncharged non-polar amino acid GLY136 with the uncharged polar amino acid SER136 renders the protein structure inflexible and may impact binding interactions, stability and conformation of the protein. For MYO3A, substitution of the positively charged and hydrophilic amino acid HIS142 with the uncharged polar and hydrophobic amino acid TRP142 could impact binding interactions and the stability of structure and conformation of the protein (Fig. 5).

### Evolutionary adaptation of human hearing: proportion of ancestral and derived alleles

Figure 4A demonstrates that the HI patients have a lower proportion of derived alleles and therefore a higher proportion of ancestral alleles at low minor allele frequencies (0·00–0·1 in Fig. 4A), suggesting that rare variants may have evolved conservatively to play a critical role in HI variable expression. The figure further shows that the control population has a higher proportion of ancestral alleles at higher minor allele frequencies. The analysis, furthermore, indicates a low proportion of ancestral alleles in *GJB2* among patients, which translates into a high proportion of derived alleles within the gene (Fig. 4B).

## Discussion

To the best of our knowledge, this is a rare study that utilizes WES to investigate HI in a SSA population. We identified very rare PLP ARNSHI variants in three known genes (*MYO3A*, *MYO15A* and *COL9A3*) (Table 2). *MYO3A* was first associated with progressive ARNSHI in a multigenerational Israeli family (31) and has since been associated with progressive HI in mice (32). A recessive form of HI with pathologic variants in *MYO3A* has also been reported in a Chinese family (33). On the other side, variants in *MYO3A* have been associated with autosomal dominant progressive HI in an African American family (34) and among individuals presenting HI from two unrelated families from the southeastern region of Brazil (35). Similar to the present study, variants in *MYO15A* were found in patients with profound HI from Morocco (36) and Tunisia (37), and variants in *MYO15A* were the most common causes (15·3%) of genetic HI in 61 Egyptian families (38). At least 200 variants have been identified in 49 of the 67 exons of *MYO15A*, which appear to be the third or fourth most common cause of severe-to-profound ARNSHI worldwide (39,40).

PLP variants in *COL9A3* were found in two patients in this study (Table 2). Variants in *COL9A3* were associated with recessive non-syndromic, moderate, progressive HI (41) as well as a recessive form of Sticker syndrome (43–45). Therefore, the two patients reported in this study will require further investigations to exclude Sticker syndrome, which is well known for its large phenotypic variations.

In this study, using WES analysis of known HI genes in this modest group of patients, we found a resolution rate of 50% (9/18). Using targeted panel sequencing of 116 HI genes, we found a higher pickup rate of 70% in a modest sample of 10 multiplex Cameroonian families in which HI was associated to *CHD23*, *LOXHD1*, *MYO7A*, *SLC26A4*, *OTOF* and *STRC* (26)*.* However, additional investigations found that the novel variants in 116 known HI genes identified in these Cameroonian families were not common in 82 unrelated isolated cases of HI of putative genetic origin (46)*.* These studies from Cameroon indicate that the extreme genetic heterogeneity (allelic and locus) of congenital HI will likely apply to populations of African ancestries. A much lower resolution rate of about 25% in 116 HI genes was observed among African Americans (24). Therefore, there is a great potential to discover novel HI genes in African populations, most likely by using multiplex families (26). The present study will contribute toward improving the much-needed data on HI variants from populations of African ancestry. Moreover, the present data could enhance the conversation on the need for more investments in genetic studies for rare and complex diseases in Africa and challenge the stereotype that, in Africa, many diseases with putative infectious or other environmental causes should not be checked for genetic causes.

Interestingly, novel population-specific variants were found in four genes (*FOXD4L6, DHRS4L2*, *RPL3L* and *VTN*) of which *RPL3L* and *VTN* indicate genetic differentiation between patients with HI and controls. Importantly, the protein products of *VTN* and *RPL3L* are shown to interact with other HI genes (Figs 2 and 3). Moreover, PPI and enrichment analysis indicated that *VTN* and *RPL3L* and their interacting proteins are significantly associated with osteoclast differentiation, which is associated with HI in osteogenesis imperfecta (Figs 2 and 3). These novel findings suggest that variants in these genes are possibly modifiers of phenotypic expression of HI. Indeed, *VTN* was a hub of a protein subnetwork along with ATPB2; the presence of a second hub protein may account for why homozygous PLP in *VTN* have not yet been associated with a human disease/condition*.*

Analysis of the fraction of ancestral alleles in the population yielded an unexpected result. Indeed, the hearing-impaired patients group presented with a higher proportion of ancestral alleles in known HI genes at low minor frequencies [the 0·00–0·10 minor allele frequency (MAF) bins]. To our knowledge, this analysis is the first to explore such an approach in HI research. This result suggests that rare variants may have evolved conservatively from the evolutionary lineage and supports the possibility of multigenic and polygenic adaptation influencing congenital HI. A point of interest is that the proportion of derived alleles in *GJB2* contrasts the generally low implication of this gene in HI in most African populations (9,17–19,21,47) and particularly in Cameroonian families segregating ARNSHI (20). This finding, that is, the high proportion of *GJB2*-derived alleles among HI patients could be due to *GJB2* being a hypervariable gene, suggests possible mutation episodes throughout its evolution. Alternatively, in some cases of congenital HI, variants in *GJB2* could be modifiers in the HI phenotypes, possibly in combination with other HI gene variants in a multigenic model. Therefore, this study supports future exploration of the possible polygenic nature of some cases of congenital HI. The possibility of polygenic HI has been previously shown using GWAS that revealed numerous suggestive loci in the most common form of deafness: adult-onset progressive HI (48–51). Future studies in a larger cohort of HI patients and controls from multiple settings in Africa are needed to validate our findings.

The present study has a few limitations: first, the modest sample size of the HI individuals. Indeed, with the extreme genetic heterogeneity, larger sample sizes are needed to capture the genetic profile that is representative of the population of Cameroon. Second, the study was performed on isolated HI cases and without the possibility of variant segregation studies within the family or in trio. Lastly, purposely designed future functional analysis should interrogate the effects of PLP on protein production and functions as well as explore the hypothesis of multigenic causes in congenital HI, using appropriate cellular or animal models. We used multiple tools instead of direct Sanger sequencing to validate variants, which is possibly another strength of this study. Indeed, studies have shown that a single round of Sanger sequencing is more likely to incorrectly refute a true-positive variant from NGS than to correctly identify a false-positive variant from NGS (52,53). Therefore, our preliminary data from an understudied African population have provided novel insights into the genomic architecture of HI.

## Conclusion

This WES investigation identified PLP variants associated with HI in 9/18 patients in three known genes: *MYO3A*, *MYO15A* and *COL9A3*. We additionally reported novel population-specific variants in four genes (*FOXD4L6*, *DHRS4L2*, *RPL3L* and *VTN*) of which *RPL3L* and *VTN* interacted with other HI genes, with *VTN* being a hub protein, suggesting a possible modifier action in the HI phenotypes. Lastly, we reported for the first time, differential frequencies of ancestral alleles versus derived alleles in known HI genes among patients versus controls. These findings may signal an evolutionary enrichment of some variants of HI genes in patients as the result of polygenic adaptation and suggest the possibility of multigenic influence on the phenotype of congenital HI, which deserves further investigations.

## Materials and Methods

### Ethical approval

The study was performed in accordance with the Declaration of Helsinki. It was granted ethics approval by the Cameroon National Ethics Committee (ethics approval N°123/CNE/SE/2010 and N°033/CNE/DNM/07) as well as the University of Cape Town Human Research Ethics Committee (ethics approval HREC REF: 455/2014, and HREC REF: 132/2010). Written informed consent was obtained from patients 18 years or older or from parents/guardians for minors, accompanied by verbal assent from the minor.

### Patients and controls

Patients were recruited from various schools for the deaf, ear, nose and throat (ENT) clinics in Cameroon. The patients were examined by qualified medical geneticists and ENT specialists. Detailed family and medical histories were obtained from the patients and their parents. In this study, 18 Cameroonian patients presenting with NSHI of either putative genetic origin (revealed by one or more affected family members or consanguinity) or of unknown origin were selected from a cohort of 582 patients (8). All the patients were tested negative for *GJB2* and *GJB6* mutations as previously reported (16,17).

The control group consisted of 129 ethnically matched individuals without personal or familial history of HI and was recruited at Yaoundé Central and Douala Laquintinie Hospitals in Cameroon.

### Genomic characterization

WES was performed at Omega Bioservices (Norcross, GA, USA) on the DNA of 18 Cameroonian patients living with HI and 129 controls from the same ethnolinguistic background. DNA concentration was quantified using the QuantiFlour dsDNA System on a Quantas Fluorometer (Promega, Madison, WI, USA); 50 ng of genomic DNA was used for library preparation following an Illumina Nextera Rapid Capture Exome kit (Illumina, San Diego, CA, USA) that uses Nextera transposomes. The libraries were then hybridized with a 37 M probe pool to enrich the sequences and were then sent for WES using the Illumina HiSeq 2500 (Illumina), 100 bp run format, with an average read depth of 30X.

### Variant calling, annotation and prioritization

The WES data was subjected to quality control using FastQC (54) and SolexaQC++ (55). The sequencing reads were aligned to hg19, build37, (56) using Burrows-Wheeler Aligner (BWA) (57,58), and local realignment was performed using Genome Analysis Toolkit (GATK) (59). Variant calling was performed using an ensemble approach, whereby GATK3.0 (HaplotypeCaller) and SamTools (60) variant callers were utilized. The final variant call set was obtained by consensus using VariantMetaCaller (61). In addition, we excluded the deletion/insertion variants with breakpoints outside the coding region.

After high confidence variants were obtained with the VariantMetaCaller, the resulting Variant Call Format (VCF) file was split into two groups: 18 HI patients and 129 controls. ANNOtate VARiation (ANNOVAR) (62) was used to independently perform gene-based annotation in the two groups in order to catalog whether SNPs caused protein-coding changes and to identify the affected amino acids. ANNOVAR was used with the ‘2015Dec18’ setting, where the population frequency for each variant was obtained on data extracted from the 1000 Genomes Project exomes and targeted exon datasets (63) and Catalog of Somatic Mutations in Cancer (COSMIC) (64). Genetic function was determined from RefGene (65,66) and different functional predictions were made from ANNOVAR’s ljb_all function. The variants set was filtered for exonic variants that were considered deleterious/damaging according to 10 different functional scores, including Sorting Intolerant From Tolerant (SIFT) (67–69), likelihood ratio test (LRT) (70), MutationTaster (71), MutationAssessor (72,73), FATHMM (74), Radial support vector machine (RadialSVM) and Likelihood Ratio (LR) (75), Combined Annotation Dependent Depletion (CADD) (76), GERP (77,78), PolyPhen2 (Poly morphism Phen otyping) (79), PhyloP and SiPhy (80). Additionally, conserved and segmental duplication sites, the dbSNP code (81) and clinical relevance reported in dbSNP138 (National Center for Biotechnology Information; (81)) were included. Thus, in the resulting annotated dataset, the variants were filtered based on whether they were: (1) rare, (2) exonic, (3) resulting in non-synonymous change, (4) resulting in a stop codon, (5) variants of known functional significance, (6) naturally selected and (7) likely deleterious (82,83). Variants that had a functional prediction status of either ‘Deleterious’ (D), ‘probably damaging’ (D), ‘disease_causing_automatic’ (A) or ‘disease_causing’(D) by all 10 functional score approaches were retained.

### Variant quality control and confirmation

We double-checked the presence and quality of variants discovered against all sample BAM files by using FastQC (54) to ensure that the reads of which the call was made or variants discovered from BAM files were of good quality. We ran FastQC on all final BAM files prior to the variant calling, then we aggregated the result from FastQC into a single report by using MultiQC (84). The summary evaluations of FastQC have resulted in six modules (Supplementary Material, Fig. S1). Furthermore, we have specifically rechecked the quality of reads in genomic regions of all our identified genes in Table 2 and generated the correlation matrix and principal components plot of all sample reads at those specific genomic regions (Supplementary Material, Fig. S2).

### Genetic differentiation and population structure

A statistical test of difference was performed to detect the possibility of unusual genetic difference between the hearing-impaired patient and control groups. The aggregated SNP frequency for all SNPs in a given gene was computed. In this regard, SNPs were mapped to their associated genes using dbSNP database and differences in the aggregate SNP frequencies were determined using Fisher’s combined probability (85).

Population structure was analyzed based on PCA using smartpca (86,87). The data from the patient and control groups were combined with data from 186 individuals of Yoruba origin (YRI) in Nigeria; 173 of Esan (ESN) in Nigeria; 280 from Western Divisions in Gambia (GWD); 116 Luhya (LWK) from Webuye, Kenya; 112 from African Ancestry in Southwest USA (ASW); 128 from Mende (MSL) in Sierra Leone and 123 of African Caribbean origin in Barbados (ACB) from the 1000 Genomes Phase3 (63). We performed a PCA the merged set of our cohort and that of seven African groups, and in a separate analysis on the merged data sets from patients and control. The first two components were plotted against each other using Genesis2.

### Fractions of ancestral and derived alleles

SNP ancestral alleles were downloaded from Ensembl and included 59 comparative alignments of 32 species (88,89). The SNPs were then checked for those present in the dbSNP database. The final patients and controls VCF files were annotated using the VCFtools ‘fillOaa’ script (90), with the ancestral allele recorded using the ‘AA’ INFO tag (89). The fraction of ancestral alleles for each SNP was computed by dividing the number of times the alternative alleles matched with ancestral alleles independently in patient and control datasets by the total number of copies of all alternative alleles across all samples for the particular SNP. The abovementioned fraction of ancestral alleles per SNP was aggregated at gene level. The fraction of derived alleles is equivalent to 1 minus the fraction of ancestral alleles. Previous studies have shown that derived alleles are more often minor alleles (<50% allele frequency) and are more often associated with risk as compared with the ancestral alleles (91).

The relationship between the fraction of derived allele and minor allele frequency in each gene was investigated. To do so, the alternative alleles were categorized into 6 bins (0–0·05, >0·05–0·1, >0·1–0·2, >0·2–0·3, >0·3–0·4, >0·4–0·5) with respect to the patient and control group frequencies, and the fractions of derived alleles in each bin were independently computed. The fraction of ancestral/derived alleles for all known and candidate HI genes was further computed. This was done by aggregating the fraction of ancestral/derived alleles at SNP-based level to gene, taking into consideration all SNPs located within the gene’s downstream or upstream region (92).

### Pathways enrichment analysis and protein–protein interactions

A comprehensive human protein–protein Interaction (PPI) network (46,92,93) was used to analyze how each of the gene variants are layered in a biological network, allowing us to extract a subnetwork. The association between gene variants in the subnetwork with human phenotypes and their potential biological pathways, processes and molecular functions was examined. This was performed using custom scripts in R (94), and the enrichment analysis was performed using Enrichr (95,96) and Panther (97,98) based on the identified gene variants.

To enable a community network analysis, the list of candidate gene variants was combined with those of the 159 known HI-associated genes (Supplementary Material, Table S1) obtained from the Deafness Variation database (12) and ClinVar (30). A clustering script in R’s igraph package (99) was used to produce a network plot, which would allow for identification of hub proteins in the subnetworks of HI genes.

### 3D protein structure prediction for functional characterization of novel variants

MD simulations were conducted to assess the effect of novel variants on protein functions. Amino acid sequences were obtained from UniProt (COL9A has UniProt protein identifier Q14050 and MYO3A has UniProt protein identifier Q8NEV4). The web server generated 1436 amino acids out of 1610 amino acids, with the missing structure of 174 amino acids on the tail end of the protein structure. The tertiary structures of the COL9A- and MYO3A-encoded proteins were generated using the I-tasser homology web server (100). All MD simulations were conducted with the GROMACS package, version 4.6.5 (101) using Amber (AMBER99SB-ILDN) force field ((102)). The system was solvated in a dodecahedron box of water. The temperature and pressure were maintained at 300 K using the Parrinello–Donadio–Bussi V-rescale thermostat (103) and a pressure of 1 kPa using the Berendsen barostat (104). The short-range non-bonded interactions were modeled using Lennard Jones potentials. The long-range electrostatic interactions were calculated using the particle mesh Ewald (PME) algorithm (105,106). The LINCS algorithm was used to constrain hydrogen bond lengths (107). Then, the velocities were assigned according to the Maxwell–Boltzmann distribution at 300 K. The equilibration of the structure NVT (constant number of particles, volume and temperature) and NPT (constant number of particles, pressure and temperature) was recorded for 10 ns each. Subsequently, a 10 ns NPT ensemble MD was conducted.

## Supplementary Material

Supplementary_Figure_1_ddaa225

Supplementary_Figure_2_ddaa225

Table_S1_ddaa225

Table_S2_ddaa225

Table_S3_ddaa225
