# Supplementary figures and images for: Whole exome sequencing reveals pathogenic variants in MYO3A, MYO15A and COL9A3 and differential frequencies in ancestral alleles in hearing impairment genes among individuals from Cameroon
Source: Hum Mol Genet. 2020 Oct 20;29(23):3729–43. doi: 10.1093/hmg/ddaa225 (PMC7861016; doi:10.1093/hmg/ddaa225)

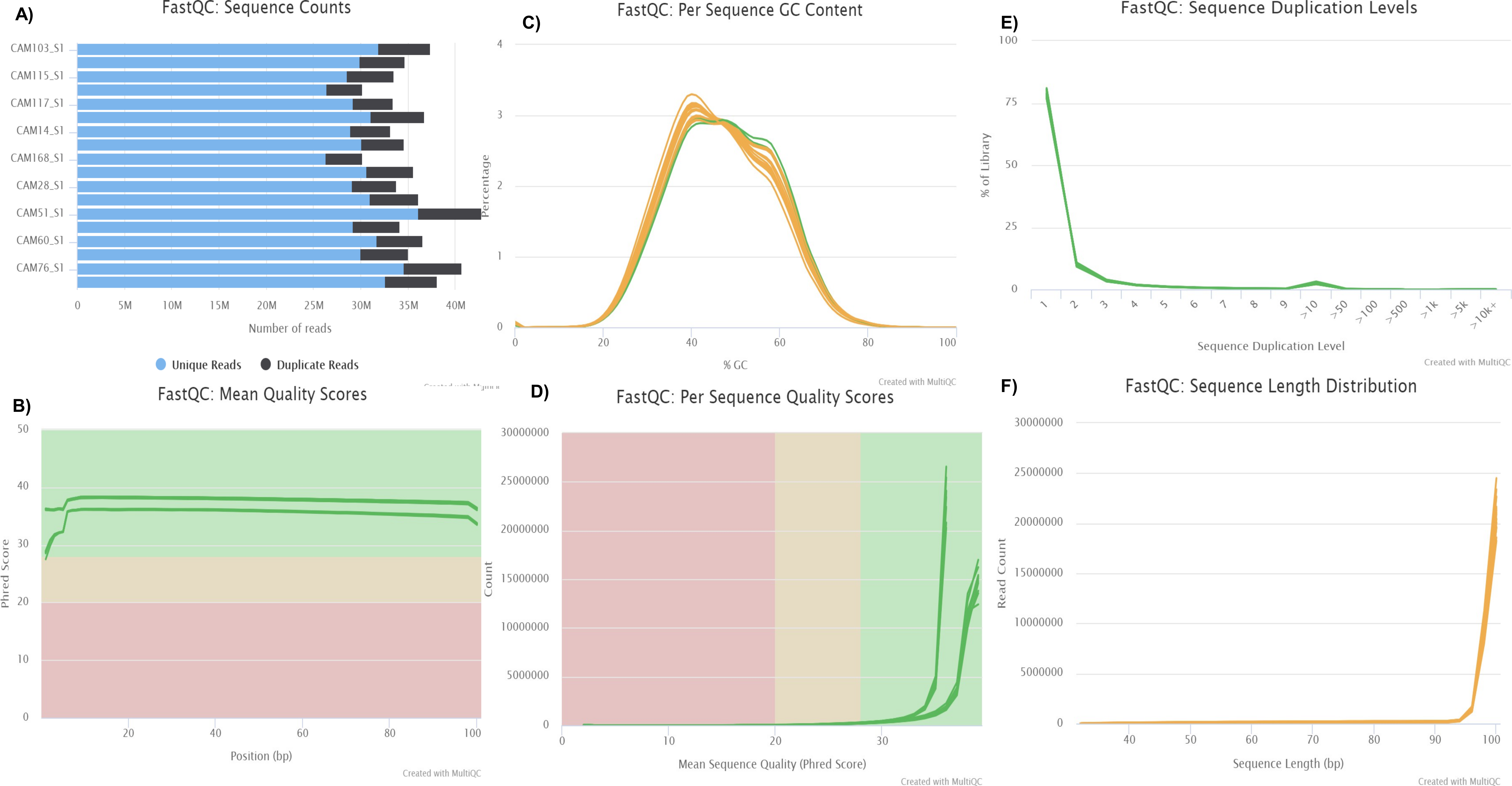

Supplement: Supplementary_Figure_1_ddaa225 [file supplementary_figure_1_ddaa225.png]

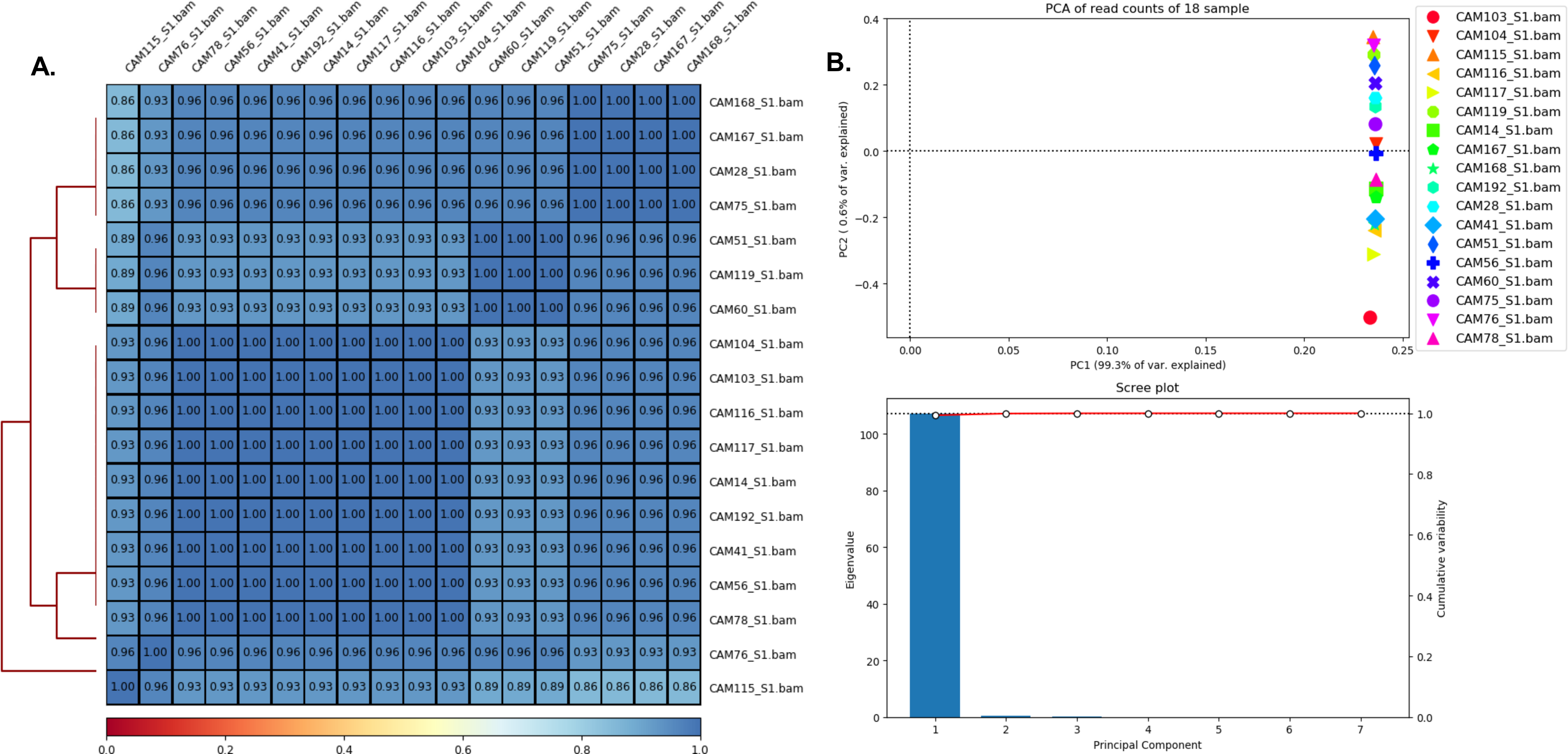

Supplement: Supplementary_Figure_2_ddaa225 [file supplementary_figure_2_ddaa225.png]
