# Supplementary material for: Whole exome sequencing reveals pathogenic variants in MYO3A, MYO15A and COL9A3 and differential frequencies in ancestral alleles in hearing impairment genes among individuals from Cameroon
Source: Hum Mol Genet. 2020 Oct 20;29(23):3729–43. doi: 10.1093/hmg/ddaa225 (PMC7861016; doi:10.1093/hmg/ddaa225)
Supplement: Table_S1_ddaa225 [file table_s1_ddaa225.docx]

**Table S1. List of 159 Known HI genes investigated**

| **Number** | Gene | **Number** | **Gene** | **Number** | **Gene** | **Number** | **Gene** | **Number** | **Gene** | **Number** | **Gene** |
| --- | --- | --- | --- | --- | --- | --- | --- | --- | --- | --- | --- |
| 1 | *ACTB* | 28 | *COL11A2* | 55 | *GIPC3* | 82 | *LOXHD1* | 109 | *PJVK* | 136 | *SOX2* |
| 2 | *ACTG1* | 29 | *COL2A1* | 56 | *GJA1* | 83 | *LRTOMT* | 110 | *PMP22* | 137 | *STRC* |
| 3 | *ADCY1* | 30 | *COL4A5* | 57 | *GJB1* | 84 | *MARVELD2* | 111 | *PNPT1* | 138 | *SUCLA2* |
| 4 | *ADGRV1* | 31 | *COL9A2* | 58 | *GJB2* | 85 | *MET* | 112 | *POLD1* | 139 | *SYNE4* |
| 5 | *AP1S1* | 32 | *COL9A3* | 59 | *GJB3* | 86 | *MITF* | 113 | *POU3F4* | 140 | *TBC1D24* |
| 6 | *ATP2B2* | 33 | *CRYM* | 60 | *GJB4* | 87 | *MSRB3* | 114 | *POU4F3* | 141 | *TBL1X* |
| 7 | *ATP6V1B1* | 34 | *DCDC2* | 61 | *GJB6* | 88 | *MTAP* | 115 | *PRPS1* | 142 | *TCF21* |
| 8 | *ATP6V1B2* | 35 | *DFNA5* | 62 | *GPR98* | 89 | *MYH14* | 116 | *PTPRQ* | 143 | *TECTA* |
| 9 | *BCS1L* | 36 | *DFNB59* | 63 | *GPSM2* | 90 | *MYH9* | 117 | *RDX* | 144 | *TIMM8A* |
| 10 | *BDP1* | 37 | *DIABLO* | 64 | *GRHL2* | 91 | *MYO15A* | 118 | *RPGR* | 145 | *TJP2* |
| 11 | *BSND* | 38 | *DIAPH1* | 65 | *GRXCR1* | 92 | *MYO1A* | 119 | *S1PR2* | 146 | *TMC1* |
| 12 | *CABP2* | 39 | *DNMT1* | 66 | *GRXCR2* | 93 | *MYO1C* | 120 | *SDHD* | 147 | *TMEM132E* |
| 13 | *CACNA1D* | 40 | *DSPP* | 67 | *HARS2* | 94 | *MYO1F* | 121 | *SERAC1* | 148 | *TMIE* |
| 14 | *CATSPER2* | 41 | *EDNRB* | 68 | *HGF* | 95 | *MYO3A* | 122 | *SERPINB6* | 149 | *TMPRSS3* |
| 15 | *CCDC50* | 42 | *ELMOD3* | 69 | *HOXA1* | 96 | *MYO6* | 123 | *SIX1* | 150 | *TMPRSS5* |
| 16 | *CD151* | 43 | *EPS8* | 70 | *HSD17B4* | 97 | *MYO7A* | 124 | *SIX5* | 151 | *TPRN* |
| 17 | *CDC14A* | 44 | *ERCC2* | 71 | *IGF1* | 98 | *NARS2* | 125 | *SLC17A8* | 152 | *TRIOBP* |
| 18 | *CDH23* | 45 | *ERCC3* | 72 | *ILDR1* | 99 | *NDP* | 126 | *SLC19A2* | 153 | *TRMU* |
| 19 | *CEACAM16* | 46 | *ESPN* | 73 | *JAG1* | 100 | *NR2F1* | 127 | *SLC22A4* | 154 | *TSPEAR* |
| 20 | *CHD7* | 47 | *ESRRB* | 74 | *KARS* | 101 | *OTOA* | 128 | *SLC26A4* | 155 | *USH1C* |
| 21 | *CIB2* | 48 | *EYA1* | 75 | *KCNE1* | 102 | *OTOF* | 129 | *SLC26A5* | 156 | *USH1G* |
| 22 | *CLDN14* | 49 | *EYA4* | 76 | *KCNJ10* | 103 | *OTOG* | 130 | *SLC4A11* | 157 | *USH2A* |
| 23 | *CLIC5* | 50 | *FAM65B* | 77 | *KCNQ1* | 104 | *OTOGL* | 131 | *SLC52A3* | 158 | *WFS1* |
| 24 | *CLPP* | 51 | *FGF3* | 78 | *KCNQ4* | 105 | *P2RX2* | 132 | *SLITRK6* | 159 | *WHRN* |
| 25 | *CLRN1* | 52 | *FGFR3* | 79 | *KIAA1199* | 106 | *PAX3* | 133 | *SMPX* |  |  |
| 26 | *COCH* | 53 | *FOXI1* | 80 | *LHFPL5* | 107 | *PCDH15* | 134 | *SNAI2* |  |  |
| 27 | *COL11A1* | 54 | *GATA3* | 81 | *LHX3* | 108 | *PDZD7* | 135 | *SOX10* |  |  |
