# Supplementary material for: Whole exome sequencing reveals pathogenic variants in MYO3A, MYO15A and COL9A3 and differential frequencies in ancestral alleles in hearing impairment genes among individuals from Cameroon
Source: Hum Mol Genet. 2020 Oct 20;29(23):3729–43. doi: 10.1093/hmg/ddaa225 (PMC7861016; doi:10.1093/hmg/ddaa225)
Supplement: Table_S2_ddaa225 [file table_s2_ddaa225.docx]

**Table S2: Candidate variants discovered in individual patients in Genes that are unlikely to explain the HI phenotype in our patients**

| Gene | Region | snp138 | A1/A2 | Genotype (Het/  Hom) | cDNA change | Protein change | Patients | Pedigree (Simplex/  Multiplex) | Cameroon controls MAF | ExAC AFR | ExAC AS | ExAC EUR |
| --- | --- | --- | --- | --- | --- | --- | --- | --- | --- | --- | --- | --- |
| *USH2A* | 1q41 | rs111033481 | C/T | Hom | c.G2546A | p.C849Y | CAM104 | Simplex | 0 | 0.0153 | 0.0006 | 0 |
|  |  |  |  |  |  |  | CAM051 | Multiplex |  |  |  |  |
|  |  |  |  |  |  |  | CAM056 | Multiplex |  |  |  |  |
|  |  |  |  |  |  |  | CAM076 | Multiplex |  |  |  |  |
| *MYO1A* | 12q13.3 | rs149803771 | C/T | Hom | c.G304A | p.E102K | CAM103 | Simplex | 0 | 0.0002 | 8.67E-0.005 | 0 |
|  |  |  |  |  |  |  | CAM056 | Multiplex |  |  |  |  |
|  |  |  |  |  |  |  | CAM075 | Multiplex |  |  |  |  |
|  |  |  |  |  |  |  | Cam076 | Multiplex |  |  |  |  |
|  |  |  |  |  |  |  | Cam078 | Multiplex |  |  |  |  |
| *HSD17B4* | 5q23.1 | rs35281104 | G/A | Hom | c.G1631A | p.R544H | Cam119 | Simplex | 0 | 0.0009 | 0 | 0 |
|  |  |  |  |  |  |  | Cam051 | Multiplex |  |  |  |  |
|  |  |  |  |  |  |  | Cam078 | Multiplex |  |  |  |  |
| Variant filtering indicated variations in USH2A, MYO1A and HSD17B4. The variations in these genes are unlikely to be causative mutations in the patients. *MYO1A* has been excluded as a hearing impairment associated gene in several prior studies (DiStefano et al, 2019). The variants in *USH2A* is defined as benign in [ClinVar](https://www.ncbi.nlm.nih.gov/clinvar/variation/48488/). Finally, the *HSD17B4* is associated with Perrault syndrome ([MIM:233400](http://omim.org/entry/233400)) and not compatible with the non-syndromic phenotype of the patients investigated. | | | | | | | | | | | | |
