# Supplementary material for: Whole exome sequencing reveals pathogenic variants in MYO3A, MYO15A and COL9A3 and differential frequencies in ancestral alleles in hearing impairment genes among individuals from Cameroon
Source: Hum Mol Genet. 2020 Oct 20;29(23):3729–43. doi: 10.1093/hmg/ddaa225 (PMC7861016; doi:10.1093/hmg/ddaa225)
Supplement: Table_S3_ddaa225 [file table_s3_ddaa225.docx]

**Table S3: Candidate Novel population specific variants (patients and controls)**

| Gene | Region | snp138 | A1/A2 | Variant Type | cDNA Change | Protein change | ExAC_AFR  MAF | ExAC_ASI  MAF | Cameroonian Controls MAF  (*N* =129) |
| --- | --- | --- | --- | --- | --- | --- | --- | --- | --- |
| *FOXD4L6* | 9p11.2 | rs369593473 | G/C | Non-synonymous | c.*2G>C | -* | 0.06186 | 0.0006 | 0.03906 |
| *DHRS4L2* | 14q11.2 | rs145720846 | G/A | Non-synonymous | c.134G>A | p.Gly45Asp | 0.003 | 0 | 0.01562 |
| *RPL3L* | 16p13. | rs143345186 | C/T | Non-synonymous | c.151G>A | p.Ala51Thr | 0.001 | 0 | 0.01923 |
| *VTN* | 17q11.2 | rs370266094 | G/T | Non-synonymous | c.719C>A | p.Pro240His | 0 | 1.501e-05 | 0.02083 |
| * Data for the SNP rs71246017 in *FOXD4L6* is not available. All the variants, including the rs71246017, occur in the exonic region of the genes and all are classified as non-synonymous. All four genes have not been previously associated with hearing impairment and each individual in the merged dataset needed to have the variant for it to filter out  Abbreviations Families’ Pedigrees, SP: simplex; MP: multiplex family; SNP: Single Nucleotide Polymorphism; ExAC: Exome Aggregation Consortium; AFR: African; AS: Asian; MAF: minor allele frequency. | | | | | | | | | |
